# *Vernonia cinerea *Less. supplementation and strenuous exercise reduce smoking rate: relation to oxidative stress status and beta-endorphin release in active smokers

**DOI:** 10.1186/1550-2783-7-21

**Published:** 2010-05-26

**Authors:** Donrawee Leelarungrayub, Sainatee Pratanaphon, Prapas Pothongsunun, Thanyaluck Sriboonreung, Araya Yankai, Richard J Bloomer

**Affiliations:** 1 Oxidative Stress and Exercise Biochemistry Laboratory. Department of Physical Therapy, Faculty of Associated Medical Sciences, Chiang Mai University, Chiang Mai, Thailand, 50200; 2 Cardiorespiratory/Metabolic Laboratory, Department of Health and Sport Sciences, University of Memphis, Memphis, TN, 38152, USA

## Abstract

**Purpose:**

The aim of this study was to evaluate the effects of *Vernonia cinerea *Less. (VC) supplementation and exercise on oxidative stress biomarkers, beta-endorphin release, and the rate of cigarette smoking.

**Methods:**

Volunteer smokers were randomly divided into four groups: group 1: VC supplement; group 2: exercise with VC supplement; group 3: exercise; and group 4: control. VC was prepared by wash and dry techniques and taken orally before smoking, matching the frequency of strenuous exercise (three times weekly). Before and after a two month period, exhaled carbon monoxide (CO), blood oxidative stress (malondialdehyde [MDA], nitric oxide [NOx], protein hydroperoxide [PrOOH] and total antioxidant capacity [TAC]), beta-endorphin and smoking rate were measured, and statistically analyzed.

**Results:**

In Group 1, MDA, PrOOH, and NOx significantly decreased, whereas TAC increased (p < 0.05). In Group 2, MDA and PrOOH decreased (p < 0.05), with no other changes noted (p > 0.05). In Group 3, MDA, PrOOH, NOx, TAC, and beta-endorphin levels increased significantly (p < 0.05). Group 4 showed no change in oxidative stress variables or beta-endorphine levels (p > 0.05). All groups had lower levels of CO after the intervention. The smoking rate for light cigarette decreased in group 2(62.7%), 1(59.52%), 3 (53.57%) and 4(14.04%), whereas in self-rolled cigarettes it decreased in group 1 (54.47%), 3 (42.30%), 2 (40%) and 4 (9.2%).

**Conclusion:**

Supplementation with *Vernonia cinerea *Less and exercise provided benefit related to reduced smoking rate, which may be related to oxidaive stress and beta-endorphine levels.

## Background

Although cigarette smoking decreased in Thailand between 1991 and 2007 from 12.2 million to 10.86 million smokers, it has increased among younger men (aged approx. 18 years) and women (aged approx. 22 years). Moreover, in low education, urban and eastern parts of the country, cigarette smoking has increased from 9.66 to 10.26 cigarettes per smoker per day [1]. Light and self-rolling cigarettes are generally used everywhere, especially in northern regions such as Chiang Mai province. Cigarette smoke contains an abundance of free radicals and prooxidant species known to negatively influence human health [2]. Increased production of free radicals from tobacco is recognized because of the more than 4,000 chemical substances found in tobacco [3]. Previous reports have noted that the levels of protein carbonyl [4] and the lipid peroxidation product malondialdehyde [5,6] are higher in smokers than non-smokers. Therefore, cigarette smoking related ill-health and disease may be mechanistically linked to increased production of free radicals. Aside from monitoring bloodborne biomarkers of oxidized molecules, evaluation of oxidative stress from smoking can be determined from exhaled hydrogen peroxide (H_2_O_2_) or carbon monoxide (CO). Nicotine in smoke is known to release neuroendocrine substances, such as beta-endorphin (β-end) [7], found in women smokers with either high doses of nicotine cigarettes or with heavy smoking [8,9]. Moreover, a previous report showed that nicotine stimulates the pituitary release of the pro-opiomelanocortin (POMC) which contains the precursor for β-end [10].

Smoking cessation has been promoted in Thailand as well as in other countries in the world. Previous evidence shows that behavioral counseling and/or pharmacotherapy is successful in long-term abstinence at a rate of approximately 30% [11,12], and pharmacotherapy is widely used in within the smoking clinic. Major disadvantages of this approach are high cost and the unwanted side effects such as nausea, dry mouth, weight gain, and sedation [13].

Exercise is a popular activity that is recognized to change in behavior and habit, including smoking cessation [14]. Acute and strenuous intensity exercise transiently increases the production of free radicals or reactive oxygen species (ROS), ultimately leading to the activation and upregulation of antioxidant enzymes following various aerobic exercise protocols [15-19]. While this increase in antioxidant defense may prove helpful in combating smoke-induce free radical producton, perhaps more importantly vigorous exercise may aid in smoking abstinenice [20]. Although moderate intensity exercise has been shown to provide both psychological benefits and improved adherence rates [20,21], heavy intensity exercise promotes reduction of tobacco withdrawal symptoms and urges to smoke [22]. Lastly, exercise has been reported to release β-end [23], especially in non-trained volunteers using strenuous exercise [24].

Vernonia cinerea Less. (VC) is classified in the Asteraceae Family as a slender stemmed plant, variable in leaf shape with pinkish-purple flowers. It has been documented and recommended in Thai traditional medicine, as in other countries, for smoking cessation, and relief of asthma, cough, fever, malaria, urinary calculi, and arthritis. Unfortunately, little scientific data are available in regards to these effects. VC is a perennial herbaceous plant and distributed in grassy areas found in Southeast Asia and Hawaii [25-27]. In a mouse model, study of the active compound in VC had noted anti-inflammatory, analgesic, and antipyretic activities [28]. In addition, a methanol extract of VC has been shown to exhibit significant anti-inflammatory activity in a rat model [29]. The active compound is proposed as a flavonoid and terpenoid [30], exhibiting both anti-oxidant and anti-inflammatory activities.

Unsing an *in vivo *study, an extract from the VC flower demonstrated an anti-oxidant effect in arthritis-induced rats by reducing lipid peroxide, and increasing the glutathione concentration in blood [31]. In humans, few scientific data are available in relation to the use of VC, in particular in regards to smoking cessation. To our knowledge, the only study of VC in relation to smoking cessation was performed at a clinic within the Thanyarak Institute, Pathumthani, Thailand. It included a 14-day VC tea supplementation program in which patients were followed for 12 weeks. Results showed a higher continuous abstinence rate (28.1%) compared to the control group (21.9%) [32]. However, they investigator did not evaluate the anti-oxidant or anti-inflammatory activities in smokers. Thus, the aim of this present study was to evaluate the efficacy of both exercise and VC supplementation alone and in combination with regards to smoking rate and blood anti-oxidant status, oxidative stress, β-end levels, over a two month period.

## Methods

### Subjects and Physical Characteristics

All volunteers participated in this study after giving their written consent. The protocol was in accordance with the 1964 Declaration of Helsinki for research on human subjects and was approved by the Ethics Committee at the Faculty of Associated Medical Sciences, Chiang Mai University, Thailand. A baseline complete blood count (CBC) was analyzed by the central laboratory at the Faculty of Associated Medical Sciences, Chiang Mai University, Thailand.

This sample included 120 Thai smoking volunteers who were addicted to nicotine in moderate to high levels, according to the Fagerstrom Test for Nicotine Dependence; (FTND) [33]. Characteristics of participants are provided in Table 1. Participants were randomized divided into four groups; group 1 (n = 30): VC supplementation; group 2: exercise with VC supplementation (n = 30); group 3: exercise only (n = 30); and group 4: usual care control--no change to normal routine (n = 30), using a block randomized allocation system. Oxidative stress status [malondialdehyde (MDA), nitric oxide (NOx), protein hydroperoxide (PrOOH), total antioxidant capacity (TAC)], and β-end concentration was determined in blood samples collected in a rested state before, after the two month intervention. Additionally, the smoking rate (cigarettes/day) was recorded.

**Table 1:** Characteristic of all smokers in four groups.

	Control (n = 28)	VC (n = 30)	Exercise plus VC (n = 28)	Exercise (n = 26)
Aged (years)	49.9 ± 9.02 (30-65)	56.1 ± 15.42 (28-82)	46.1 ± 11.35 (28-73)	49.1 ± 15.9 (28-87)
BMI (kg.m^-2^)	21.05 ± 1.56 (19.45 - 24.45)	22.07 ± 1.53 (18.55-25.71)	23.45 ± 2.23 (20.45-25.25)	22.24 ± 1.37 (20.08-25.71)
Smoking rate (cigarette/day)				
5-10 cigarettes	18	21	13	12
11-20 cigarettes	10	9	15	14
Nicotine score	7.09 ± 1.15 (5-9)	7.17 ± 1.76 (5-10)	7.56 ± 1.02 (5-10)	7.00 ± 1.88 (5-10)

### *Vernonia cinerea *Less. Preparation

Naturally grown,raw VC was collected from local clean area which uses natural growth without spray of insect-toxin drugs at Chiang Mai Province, Thailand. VC was washed four times and cut to small piece approximately one inch and heated until dry by an oven at 70 decree C. VC was then kept in a sterile bottle which contained a small bag of anti-moisture silica-gel pills. Each day for preparation, 20 grams of dry VC material and 3 cups of clean water (390 milliliter) was boiled in a traditional pot until water evaporated to 1 cup (130 milliliter). Condensed VC juice was then preserved in a clean bottle and was provided to subjects to drink prior to smoking each, three days per weeks for two months.

### Exercise program

The exercise program was performed on treadmill at the local community center, short warm up was performed by stretching the upper and lower limbs for approximately 3. The actual exercise consisted of 30 min of running with a progressive incline and speed program, with a maximum intensity of 85% of maximal heart rate (calculated manually by a trainer). The Rate of Perceived Exertion (RPE) was limited to under 15 or hard exertion (6-20 Borg Scale) [34]. Our objective was to endure that participants were performing strenuous exercise. The session concluded with 3 min of slow speed walking. Each exercise session was monitored by research personnel or a village health volunteer.

### Malondialdehyde assay

The protocol for MDA was modified from the Leelarungrayub's protocol [35]. 250 μl of plasma was mixed with 750 μl of ortho-phosphoric acid (2.5%, *v:v*) and vortexed. Then, 500 μl of TBA (0.2 mol/L) in Tris solution (0.14 mol/L) was added. After incubation in a water bath (90°C) for 30 min, all samples were cooled and centrifuged at 10,000 rpm for 3 min. A clear pink color of supernatant was read with a spectrophotometer at 532 nm. The yield of MDA in the sample was calculated by comparing with the absorbance of standard Tetramethoxypropane (TMP) (Sigma) (0-50 μmol/L).

### Nitrite assay

Plasma nitrite was evaluated as an indirect marker of NOx, using Griess reagent following Promega's Instructions for use of the Griess reagent system [36]. First, 200 μl of plasma were mixed with 500 μl of 0.1% of *N*-1-napthylethylenediamine dihydrochloride (NED) in water and left in the dark for 5 min, then 500 μl of 1% sulfanilamide were added to 5% phosphoric acid and kept in the dark again for 5 min. A slightly pink color was produced with an absorbance reading at 520 nm. Nitrite in plasma was calculated by comparing with the absorbance of standard sodium nitrite (NaNO_3_) (0-40 μmol/L).

### Protein hydroperoxide assay

The protocol for PrOOH was modified from that of Gay et al (2003) [37]. Plasma protein at 200 μl was precipitated with 0.5 mol/L perchloric acid (PCA) and resolved with 700 μl of guanidine hydrochloride (GuHCL) (6 mol/L). Then, 40 μl of 0.2 mol/L of perchloric acid, 25 μl of xylenol orange (5 mmol/L), and 10 μl of ferrous solution (5 mmol/L) were added. The whole mixture was left in the dark for 30 min before being centrifuged at 10,000 rpm for 3 min. The yellow supernatant was read for absorbance at 560 nm. The level of PrOOH was calculated by comparing with the standard *tert*-butyl hydroperoxide (0-10 μmol/L).

### Total antioxidant capacity assay

Total antioxidant capacity of fresh plasma was assayed with ABTs cation radical decolorization [38]. Stock ABTs cation radical was produced by mixing ABTS (14 mmol/L) and potassium persulfate (14 mmol/L) together and leaving in the dark overnight. Working ABTs cation radical was diluted from stock ABTs with deinoized water, until absorbance at 734 nm was shown at 0.7 ± 0.02 before adding plasma. The 10 μl of plasma was added to 990 μl of working solution ABTs cation radical in a plastic cuvette (size 1.5 ml), and gently shaken 9 times before adding again in the spectrophotometer. Decreased absorbance was recorded continuously every 1 min for 3 minutes, and finally calculated to ΔA/min. Total antioxidant capacity (TAC) of plasma was calculated by comparing with the ΔA/min of standard Trolox (0-10 mmol/L) at 0.1.

### Beta-endorphin assay

The protocol for evaluation of β-end in plasma was performed according to the guidelines in β-end ELISA kit (Catalog Number EDRF.96, MD Biosciences, Inc. USA). 500 μl of plasma was acidified with 500 μl of 1% trifluoroacetic acid (TFA) and mixed, then centrifuged at 10,000 × g for 20 min at 4degrees C. We then equilibrated a SEP-Column (200 mg of C18) by washing with 60% acetonitrite in 1% TFA (1,000 μl) followed 3 times with 1% trifluoroacetic acid (3000 μl). We loaded the acidified plasma solution onto the pre-treated C-18 SEP- Column, slowly washed the column with 1% trifluoroacetic acid and collected eluant. We evaporated the eluant to dryness in a centrifugal concentrator and collected this in a polypropylene tube and kept he dried sample at -20 degress C. In the ELISA system, the dried sample was reconstituted with assay buffer and a 50 μl of sample, 25 μl of primary anti-serum, and 25 μl of biotinlyated β-end was loaded into each wells. After incubation for 2 hr at room temperature, wells were washed washed three times, and dried. We then added 100 μl of diluted SA-HRP solution in each well, except for the blank, and incubated for 1 hr at room temperature. The plate was washed again three times and dried. Finally, we added 100 μl of TMB solution to each well, and incubated for 1 hr at room temperature. The reaction was stopped with 2N HCL and absorbance read at 450 nm. The concentration of β-end was calculated with the standard curve of standard β-end (0.01-1,000 ng/mL).

### Measurement of end-expiratory CO level

For a measure of exhaled carbon monoxide (CO), CO was evaluated with a MicroCO (MC02, Micro Medical Limited, UK). All smokers were standing during test. Subjects were instructed to, hold inspired air for 10-15 seconds, and then expire slowly until evacuating the end-expiratory air. Three repetitive measurements were performed confirm values, and we recorded the maximal level of CO (ppm).

### Statistic analysis

All parameters are reported as the mean (SD). A multiple variables repeated measurement with a Linear model analysis (4 groups × 2 time) was used for statistical analysis. The significance was set at p = 0.05.

## Results

Participant characteristics for those subjects completed all phases of the study are presented in Table 1 and are as follows: group 1, VC supplementation (n = 30) (mean aged = 56.1 ± 15.42 years); group 2, exercise with VC supplementation (n = 28) (mean aged = 46.1 ± 11.35 years); group 3, exercise only (n = 26) (mean aged = 49.1 ± 15.9 years); and group 4, a control (n = 28) (mean aged = 49.9 ± 9.02 years). For all smokers, the baseline CBC results showed values within normal range (7.56 ± 1.78 x103 cc.mm in WBC, 14.2 ± 2.34 g/dl in Hb, 45.68 ± 2.43% in Hct, 233.56 ± 58.32 × 105 in Plt, 55.31 ± 5.94% in Neutrophil, 32.50 ± 10.14% in lymphocyte, 2.56 ± 3.16% in monocyte, and 0.76 ± 0.62% in basophil respectively). Mean BMI of all groups were within the normal criteria of normal levels (18.5-24.9 kg m^-2^) according to the ACSM'Health-related physical fitness assessment manual [39]. Basic data showed that the smoking rate (cigarettes per day) was 5-10 (n = 21) and 11-20 (n = 9) in group 1, 5-10 (n = 13) and 11-20 (n = 15) in group 2, 5-10 (n = 12) and 11-20 (n = 14) in group 3, and 5-10 (n = 18) and 11-20 (n = 10) in group 4 (Table 1).

### Smoking rate (cigarettes per day)

**Figure 1:**
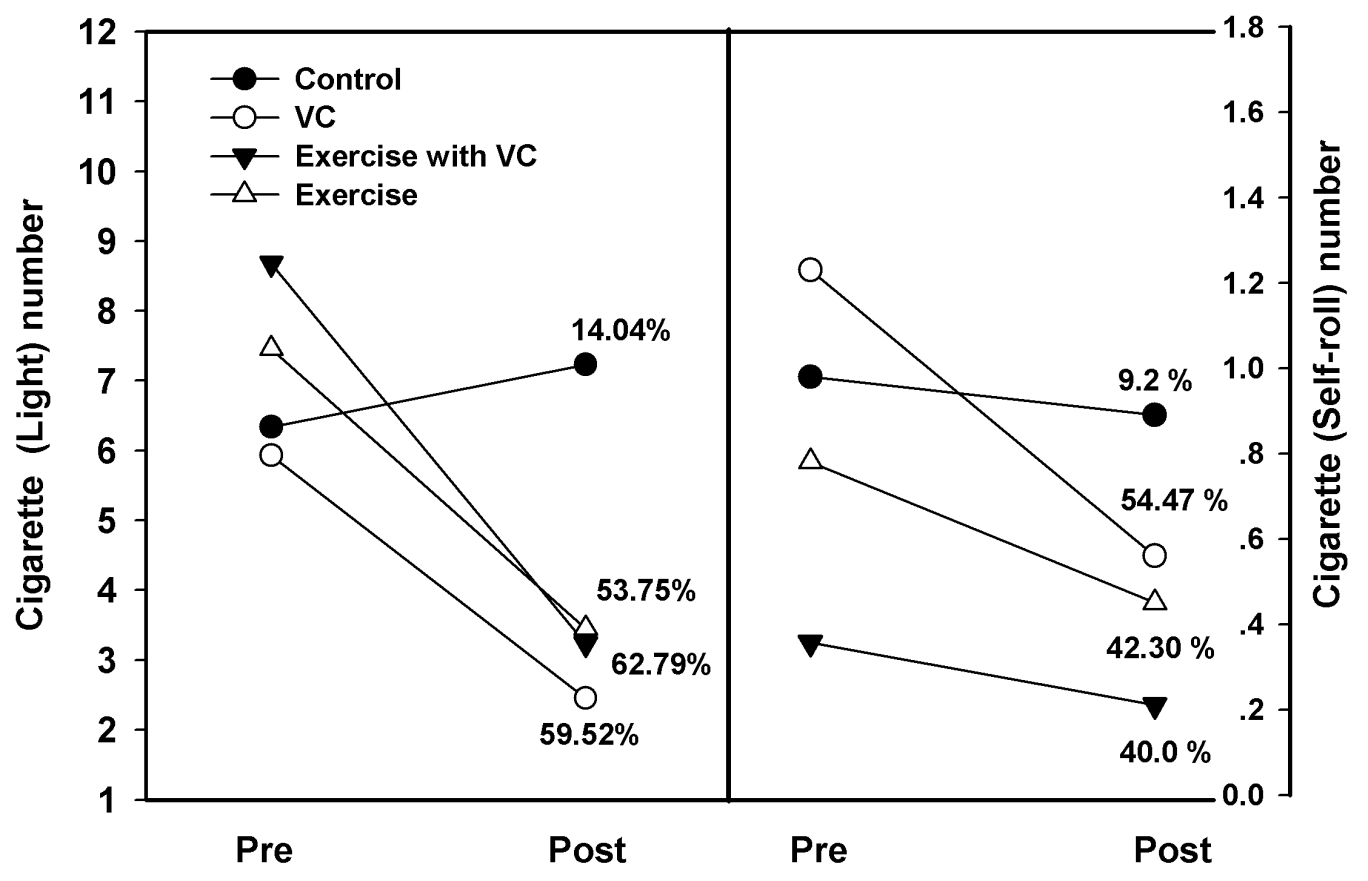
**Cigarette yields per day of light (right) and self-rolling (left) types between pre- and post-intervention periods in each groups, control, VC, exercise with VC, and exercise**. Each point represents the mean of cigarette yield per day. The percentage at post-intervention was compared to the pre-intervention.

In this study, the cigarettes were divided into two types, light and self-rolled. In Figure 1, the yields of light and self-rolled cigarettes at the pre-intervention period were (5.93 ± 3.21 via 1.23 ± 2.01) in group 1, (8.68 ± 5.21 via 0.35 ± 2.34) in group 2, (7.46 ± 6.23 via 0.78 ± 1.11) in group 3, and (6.34 ± 2.20 via 0.98 ± 1.23) in group 4. After 2 months of intervention, results showed that the yield of cigarettes per day had reduced significantly to lower than that at the pre-intervention period in all groups, excepted group 4. The findings were 2.45 ± 4.67 (p < 0.05) via 0.56 ± 2.34 (p < 0.01) for group 1, 3.23 ± 4.32 (p < 0.01) via 0.21 ± 1.23 (p < 0.05) for group 2, 3.45 ± 2.21, (p < 0.01) via 0.45 ± 2.89, (p < 0.05) for group 3, and 7.23 ± 2.34 via 0.89 ± 1.34 for group 4 (p > 0.05). When calculating the percentage of cigarette reduction per day for both light and self-rolled types, it was reduced in all groups, excepted for group 4. Reduction values of (59.52%, and 54.47%) for group 1, (62.79%, and 40.00%) for group 2, (53.75%, and 42.30%) for group 3. A 14.04% increase (light) 9.2% reduction (self-rolled) was noted for group 4.

### Oxidative Stress Biomarkers

**Figure 2:**
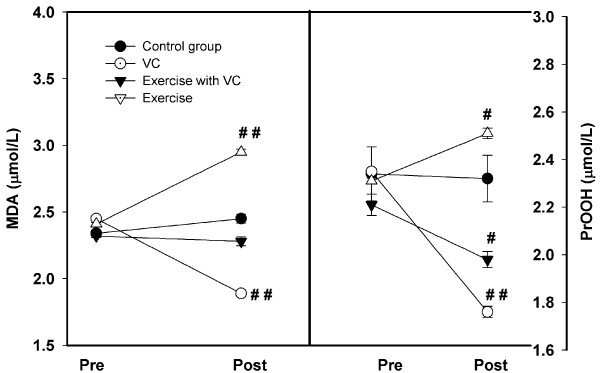
**The levels of MDA (left) and protein hydroperoxide (PrOOH) (right) between pre- and post-intervention periods in each group, control, VC, exercise with VC, and exercise only**. Each point represented the mean and standard deviation and significant level at p < 0.05 (#) and p < 0.01 (##).

At the pre-intervention assessment, MDA and PrOOH were not difference between groups (Figure 2). The MDA levels of all groups had no significant difference, i.e. group 4 (2.34 ± 0.023 μmol/L), group 1 (2.45 ± 0.018 μmol/L), group 2 (2.32 ± 0.012 μmol/L), and group 3 (2.41 ± 0.023 μmol/L). After the two month intervention, the results showed a significant decrease in MDA for group 1 (1.89 ± 0.023 μmol/L, p < 0.01), and a slight non-significant decrease in group 2 (2.28 ± 0.034 μmol/L), whereas, a significant increase occurred in group 3 (2.95 ± 0.02 μmol/L, p < 0.05), and a slight increase in group 4 (2.45 ± 0.034 μmol/L)

As for PROOH, group 4 showed unchanged PrOOH levels (from 2.34 ± 1.11 to 2.32 ± 0.98 μmol/L), whereas, group 3 showed slightly increased levels (from 2.31 ± 0.01 to 2.51 ± 0.22 μmol/L). The levels of PrOOH decreased in group 1 (2.35 ± 0.67 to 1.76 ± 0.23 μmol/L) and group 2 (2.21 ± 0.04 to 1.98 ± 0.03 μmol/L) during the two month intervention period (p > 0.05).

**Figure 3:**
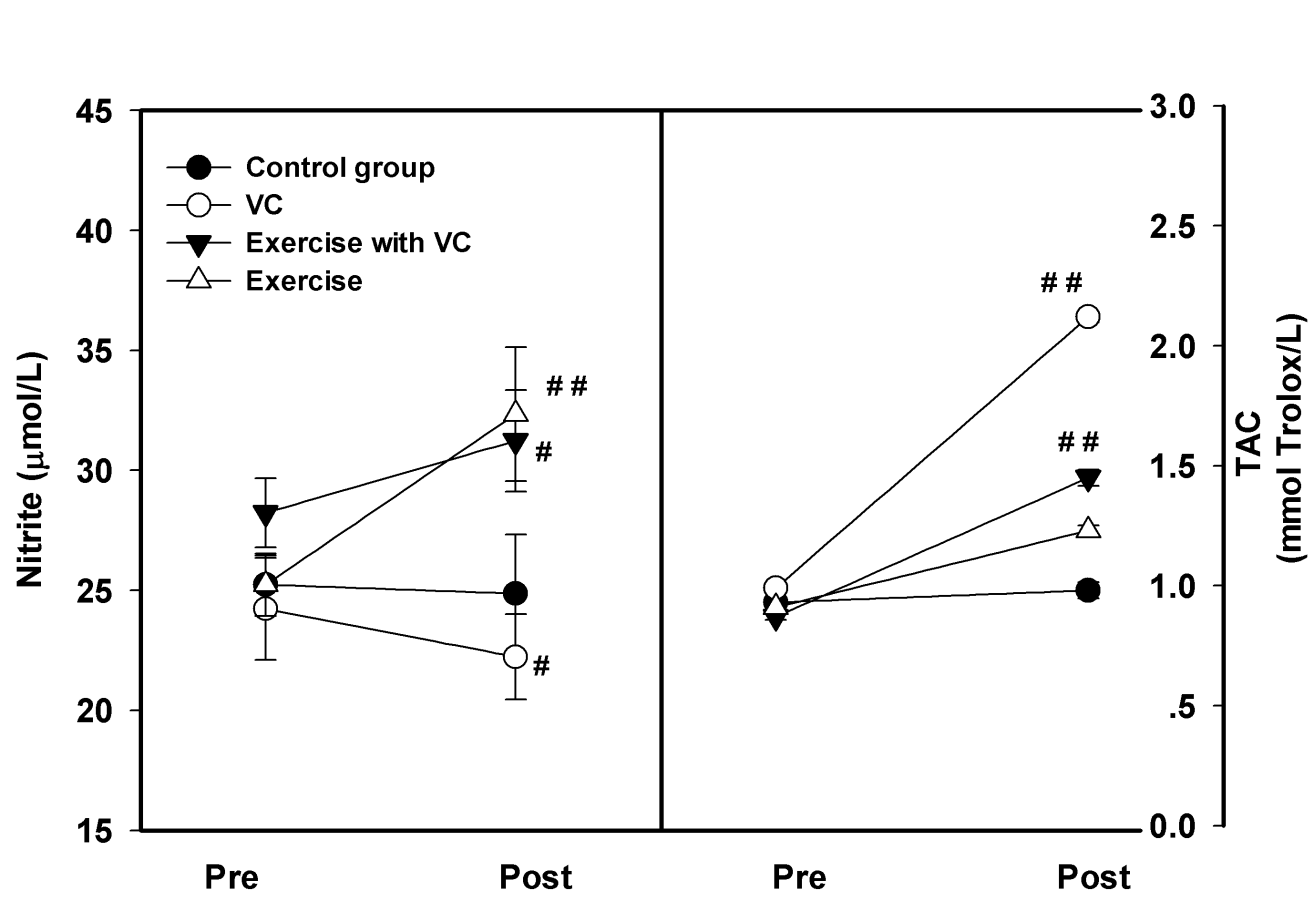
**The levels of nitrite (left) and Total antioxidant capacity (TAC) (right) between pre- and post-intervention periods in each group, control, VC, exercise with VC, and exercise only**. Each point represented the mean and standard deviation and significant level at p < 0.05 (#) and p < 0.01 (##).

For nitrite (Figure 3, left), a significant decrease was shown in group 1 (22.23 ± 1.78 μmol/L), compared to pre-intervention (24.23 ± 2.12 μmol/L). However, group 3 showed a significant increase (32.34 ± 2.78 μmol/L) compared to pre-intervention (25.23 ± 1.30 μmol/L), as did group 2, but at a lower level (31.23 ± 2.12 μmol/L), compared to pre-intervention (28.23 ± 1.45 μmol/L). On the other hand, group 4 showed no significant change (24.87 ± 1.28 and 25.23 ± 1.11 μmol/L) (p > 0.05).

In the TAC status (Figure 3, right), in all groups after two months intervention, the levels of TAC improved significantly in group 1 (2.12 ± 0.012 mmol/L), group 2 (1.45 ± 0.034 mmol/L), and group 3 (1.23 ± 0.012 mmol/L), compared to pre-interventine (0.99 ± 0.012, 0.87 ± 0.013, 0.91 ± 0.011 mmol/L, respectively), but they did not change in group 4 (0.93 ± 0.023 and 0.98 ± 0.031 mmol Trolox/L) (p > 0.05).

### Exhaled CO and β-Endorphin levels

**Figure 4:**
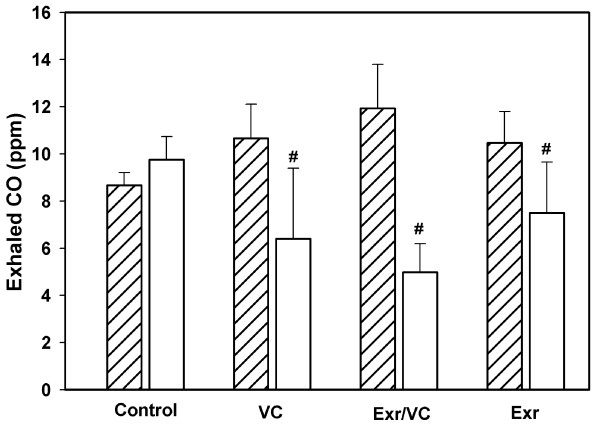
**The exhale carbon monoxide (CO) between pre- (oblique line) and post-(no line) intervention periods in each group, control, VC, exercise with VC, and exercise only**. Each bar represented the mean and standard deviation, and significant level at p < 0.05 (#).

**Figure 5:**
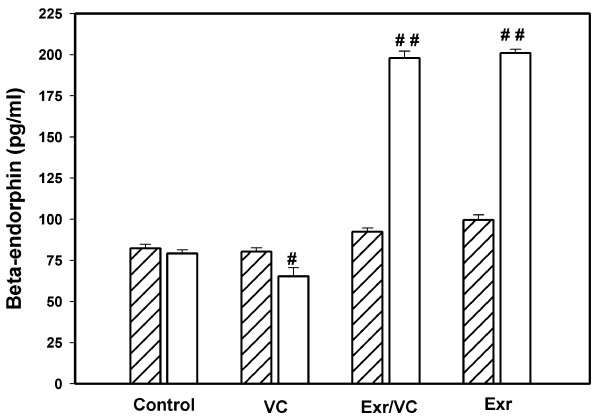
**The beta-endorphin levels between pre- (oblique line) and post-(no line) intervention periods in each group, control, VC, exercise with VC, and exercise only**. Each bar represented the mean and standard deviation, and significant level at p < 0.05 (#) and p < 0.001 (# #).

This study found that the exhaled CO level (Figure 4) significantly decreased in group 1 (5.40 ± 2.99 ppm, p < 0.01), group 2 (4.98 ± 1.22 ppm, p < 0.01), and group 3 (4.96 ± 2.15 ppm, p < 0.01), compared to pre-intervention (10.66 ± 1.45, 11.93 ± 1.87, 10.46 ± 1.33 ppm), whereas, group 4 showed a slight increase (8.67 ± 1.11 and 9.75 ± 1.28 ppm). For β-end concentration (Figure 5), group 2 (198.00 ± 4.23 pg/ml) and group 3 (201.00 ± 2.31 pg/ml) improved significantly compared to base line (92.45 ± 2.12 and 99.50 ± 3.23 pg/ml) (p < 0.001), whereas, reduction was significant in group 1 (65.23 ± 5.23 pg/ml) compared to base line (80.23 ± 2.45 pg/ml) (p < 0.05). There was no change in group 4 between baseline (82.34 ± 2.34 pg/ml) and after the two month intervention (79.23 ± 2.12 pg/ml).

## Discussion

From these results, it can be concluded that VC supplementation in a small group of smokers can reduce oxidative stress and the rate of cigarette smoking per day, but VC had no effect on the β-end level. Exercise was chosen for changing behavior to smoking cessation and improved β-end level, but our heavy intensity of exercise actually increased oxidative stress. It is possible that a lower intensity exercise program may be best in this regard, unless the exercise is combined with supplementation such as VC. The combination of VC supplementation and exercise helped to reduce the rate of smoking when compared to a control group, especially in smokers using light cigarettes, whereas the combined intervention also improved both β-end levels and antioxidant status as measured by TAC

### *Vernonia Cincerea *and smoking cessation

From this preliminary study, we note very interesting results of the co-intervention between the natural plant, VC and strenuous exercise, in relation to smoking rate in a local northern area, Chiang Mai province, Thailand, which may have application to her parts of South-East Asia or Hawaii [25-27]. Previous study in a short 14 day clinical trial at Thanyarak Institue, Pathumthani in Thailand [32] found a higher continuous abstinence rate (28.1%) in a VC supplemented group, compared to a control group of smokers (21.9%). However, the design of the study was difference from ours, with regards to the preparation of the VC juice, as well as the intake of the juice. In the present study, our subjects were asked to drink the VC juice prior to each time they planned on smoking. They were instructed to keep the condensed VC juice in the mouth for 1-2 seconds prior to being swallowed.

In our initial experiences with VC, all smokers had adverse events with tongue bitter or numbness (100%), with a few having nausea (10.5%), and headache (5.2%); however, this ceased after the initial week of treatment. Whereas in the study of Wongwiwaathananukit and co-worker [32], subjects reported more adverse events such as tongue numbness (46.9%), upper abdominal pain (21.9%), nausea (28.1%), headache (40.6), palpitation (15.6%), drowsiness (59.4%), craving reduction (59.4%), and dislike for the taste and smell of cigarette smoke (62.5%) after 14 days of VC supplementation.

### Baseline of Oxidative Stress

This study showed relative high oxidative stress values in all smokers at the pre-intervention period. Previous study by Bloomer and colleagues [40] in young smokers (24 ± 4 years) showed lower MDA (0.919 ± 0.32 μmol/L) than our study (more than 2.0 μmol/L), which included middle to older aged subjects (46.1 ± 9.02 years in control, 56.7 ± 15.42 years in VC, 46.2 ± 11.35 years in exercise with VC, and 49.5 ± 15.9 year in exercise).However, the TAC levels appeared similar.

For NOx with nitrite levels in all smokers; 25.23 ± 1.11 in control, 24.23 ± 2.12 μmol/L in VC, 28.23 ± 1.45 μmol/L in exercise with VC, and 25.23 ± 1.30 μmol/L in exercise (Figure 3 left). Previous study in healthy, sedentary, younger (22.5 ± 3.45 years) or older individuals (65.7 ± 6.14 years) noted mean levels lower levels which were slightly lower but similar to our values (23.78 ± 5.72 μmol/L and 22.17 ± 6.14 μmol/L) [41]. The higher nitrite levels in our study may be related to the high level of PrOOH (Figure 2 right). Many reports show that NOx can react and damage protein. For example, Ischiopoulos and al-Mehdi [42] showed that peroxynitrite was generated by the reaction of NOx with superoxide and has a direct effect on tryptophan and cysteine, including protein fragmentation. Previous study in smokers showes the high level of oxidized protein compared to nonsmokers [43].

### Intervention: Oxidative Stress

Oxidative stress values changes with the intervention in all groups except for group 4. In Group 1, MDA, PrOOH, and NOx significantly decreased, whereas TAC increased. In Group 2, MDA and PrOOH decreased, with no other changes noted. In Group 3, MDA, PrOOH, NOx, TAC, and beta-endorphin levels all increased significantly

Figure 3 shows the plasma NOx levels after the 2 month intervention, and results showed an improved NOx level in group 3 (32.34 ± 2.78 μmol/L) and a slightly increased level in group 2 (1.23 ± 2.12 μmol/L), but it was lower than in a previous study by Franco [41], which showed higher levels of NOx in both healthy younger (44.73 ± 6.48 μmol/L) and older subjects (45.88 ± 9.84 μmol/L). Physiologically, a lower level of NOx can be indicative of a depressed function in nitric oxide synthase (NOS) and lower release of NOx in the smoker's plasma, which can cause hypertension or stroke in the long term [44]. Fortunately, results in our study showed an increasing level of NOx in group 2 and 3, which might aid overall cardiovascular health. We also noted improvement of TAC (statistically) in all groups, excepted group 4 (VC > exercise and VC > exercise, alone). A previous study showed the antioxidant activity of VC flowers in arthritis-induced rats [31], which corresponded to a reduction in lipid peroxide in the liver, plasma and spleen, and also an increase in glutathione in the blood.

### Intervention: β-endorphin and CO

Although this study was carried out in a small group of smokers, the results related to β-end showed a significant increase after strenuous exercise (Figure 5). The β-end level in this study was nearly the same as the mean value (79.46 ± 6.31 pg/ml) of a previous study [45] of smokers who consumed less than 10 cigarettes per day. In all the smokers of our study, the mean number of cigarettes smoked per day was less than 10; group 4 (6.34 ± 2.20), group 1 (5.93 ± 3.21), group 2 (8.68 ± 5.21), and group 3 (7.46 ± 6.23). The β-end levels were 82.34 ± 2.34 pg/ml (group 4), 80.23 ± 2.45 pg/ml (group 1), 92.45 ± 2.12 pg/ml (group 2), and 99.50 ± 3.23 pg/ml (group 3). After the 2 month intervention, only group 2 (198.00 ± 4.23 pg/ml) and 3 (201.00 ± 2.31 pg/ml) showed a significant increase in β-endorphin levels. It should be noted that in group 1, the slight reduction of β-end level was significant (p < 0.05), thus suggesting that the increased β-end in group 2 and 3 most likely resulted from exercise only and not from VC. From previous reports, the intensity and type of exercise for increasing β-end is still unclear, but resistance and moderate intensity exercise did not influence β-endorphin level [46].

There has been little evidence to support a specific exercise program for smoking cessation or for reducing the rate of smoking. A previous study of 10 women smokers (27 ± 11 cigarettes per day and 29 ± 15 ppm of exhaled CO) by Marcus and co-worker [23] showed that a smoking cessation program, plus exercise via cycle ergometery at 70-85% intensity for three supervised sessions per week for 15 weeks, resulted in 30% smoking abstinence at the end of treatment. However, in our study, the rate of cigarette consumption was lower than 10 cigarettes per day, with lower CO (Figure 4). Thus, the reduction rate was higher for light smoking (62.79% in group 2, 59.52% in group 1, 53.75% in group 3) than self-rolled cigarettes (54.47% in group 1, 42.30% in group 3, 40.0% in group 2) (Figure 1).

### VC and Exercise for smoking cessation

Vigorous exercise has been used to assist in smoking cessation, as this results in increased caloric expenditure [47], which may offset the often observed weight gain associated with cessation and can also serve as a substitute behavior during cessation trials [48]. Exercise may be associated with positive, mood changes [49], which aid in decreasing both physiological [50] and psychological [51] conditions and is recommended for long-term sucess in smoking cessation [52].

The active compounds in VC have been rarely studied, in human subjects in particular. It is therefore noteworthy that the flower of VC extract contains refluxing 80% ethanol, active compounds composed of various substances as steroids, saponins, alkaloids, carbohydrates, flavonoids, phenols, tanins, and proteins [31]. Currently, the work of Misra and co-worker [53] shows that extracts of the VC leaf include chloroform, methanol, and petroleum ether, which have analgesic, antipyretic, and anti-inflammatory activities in a rat model. It has been suggested that VC decreases locomotory, exploratory behavior and increases the body scratching behavioral model that is probably due to CNS depression with excitatory activities of the monoamines neurotransmitters [54,55]. However, there is no evidence supporting this hypothesis in regards to smoking behavior. In our study, the active compounds in VC juice were not analyzed; hence, we cannot state with confidence the chief actives responsible for teh reduction in smoking rate. Plans for further analysis of VC in future experimentals should be made.

## Conclusion

This is a preliminary study of the influence of VC supplementation and exercise on oxidative stress and β-end release, with relevance to smoking cessation. Results indicate the use of VC supplementation for reducing smoking rate,with and with our exercise. The reduction in smoking rate may be associated with levels of oxidative stress. Both VC supplementation and exercise may compensate for nicotine addiction. Additional studies using larger samples, as well as a combination of both men and women who are heavy smokers are needed to extend these findings.

## Competing interests

The authors declare that they have no competing interests.

## Authors' contributions

DL was responsible for obtaining funding, designing the study, establishing community connections, performing laboratory testing, and performing data analysis. AY and TS performed data collection. SP and PP assisted with data collection and in establishing community connections. Richard J Bloomer assisted with manuscript writing and preparation. The final manuscript was read and approved by all authors.
